# Molecular docking analysis of flupenthixol and desmethylastemizole with the apoptotic regulator proteins CFLAR and TRAF2 linked to lung carcinoma

**DOI:** 10.6026/97320630017470

**Published:** 2021-04-30

**Authors:** Subrata Das, Anupam Das Talukdar, Rajat Nath, Deepa Nath, Ashikur Rahaman, Shamee Bhattacharjee, Manbendra Dutta Choudhury

**Affiliations:** 1 Department of Life Science and Bioinformatics, Assam University, Silchar-788011, India; 2 Department of Zoology, West Bengal State University, Berunanpukuria, Malikapur, North 24 Parganas, Barasat, Kolkata-700126, West Bengal, India; 3 Department of Botany, Gurucharan College, Silchar 4, Assam, India

**Keywords:** Molecular docking, CFLAR, TRAF2, apoptosis, lung carcinoma, mutation

## Abstract

It is known that molecular changes in apoptotic genes due to mutation may cause disruption of apoptotic pathway resulting in an abrupt increase in cell proliferation. Therefore, it is of interest to identify compounds that could potentially replenish the changes in the apoptotic pathway, resulted from mutation. The gene network analysis using the Network Analyzer Plugin of Cytoscape (3.5.1) shows CFLAR and TRAF2 as influential genes in the apoptotic pathway. Mutation in these genes brings loss in apoptotic property of a cell and thus increases the cell proliferating activity. Thus, data on the molecular docking analysis of four natural compounds from Ottelia alismoides (L.) Pers with the two target proteins were reported. Flupenthixol and desmethylastemizole was found to be two efficient ligand molecules based on ligand-target interaction. In stereochemical quality assessment, the Ramachandran plot analysis of receptors indicates the better stereochemical characteristics for receptor-ligand interaction.

## Background:

Cancer is one of the deadliest complications worldwide. Based on the latest report of World Health Organization (WHO), approximately 9.6 million deaths occurred globally in 2018 is due to cancer and is estimated to rise to 20 million new cases by 2025 [1]. Lung cancer is more than one-fourth of deaths worldwide. Nearly sixty percent of lung cancer patients are diagnosed at late stages with metastasis, where cell proliferate very quickly, and chances of survival become very minimal. Thus, identification of novel therapeutic targets and lead molecule against lung cancer proliferation and metastasis are urgently essential to enhance patients survival [2]. Apoptosis has been known to play a vital role in the maintenance of tissue homeostasis by selective eradication of unwanted cells. Genetic changes or mutation resulting in loss of apoptosis or apoptosis-signaling pathways in the distorted cells are likely to be key factors of carcinogenesis. Furthermore, introduction of apoptosis of cancer cells is identified as a useful tool for cancer treatment [3]. Network pharmacology is a modern subject of interest based on the features of biological molecules. It provides an insight into multiple influential databases, which allows us to understand the underlying mechanisms of medicine and diseases. Therefore, it is of interest to document the molecular docking analysis data of Flupenthixol and desmethylastemizole with the apoptotic regulator proteins CFLAR and TRAF2 linked to lung carcinoma [4] for consideration in drug discovery.

## Materials and methods:

### Testing of Hypothesis:

**Figure 1:**
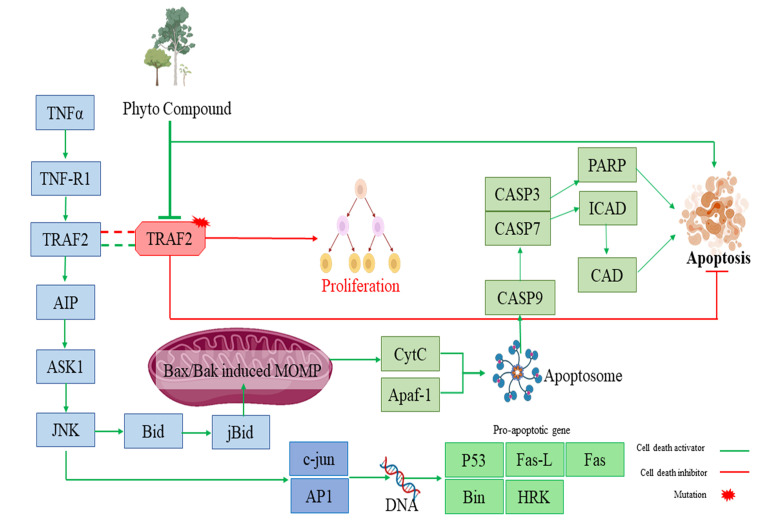
Pathway depicts the regulation of TRAF2 in normal state of apoptosis and in abnormal state of proliferation

**Figure 2:**
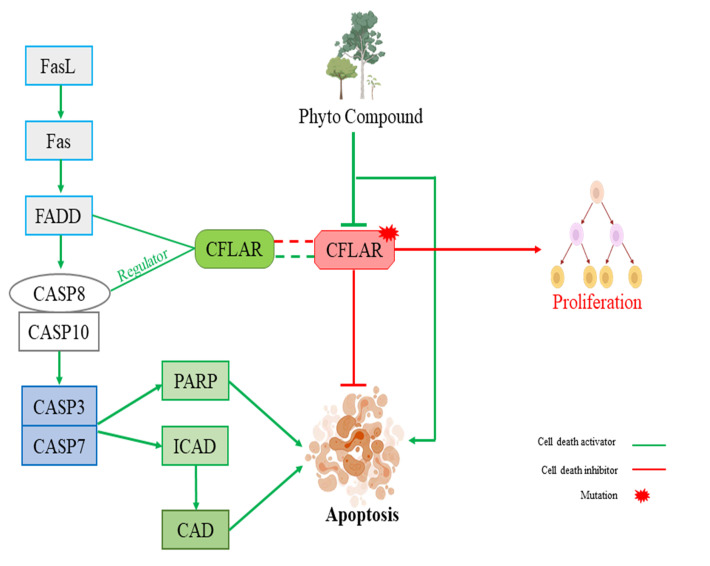
Pathway depicts the regulation of CFLAR in normal state of apoptosis and in abnormal state of proliferation

TRAF2 and CFLAR are two important genes in the apoptotic pathway and they play a vital role in proper functioning of apoptosis in cell. Certain cases of mutation in TRAF2 and CFLAR due to carcinogenesis or mutagenesis, proliferation takes place. At that condition ligand molecule binding with the TRAF2 (Figure 1) and CFLAR (Figure 2) can block their activity to minimize their gain of proliferating nature. This activity may help the other influenced gene of the apoptotic pathway to normalize or replenish their active apoptotic pathway.

### Validation of Hypothesis:

Gene Network Analysis:

Genes involved in the apoptotic process were retrieved from the Kyoto Encyclopedia of Genes and Genomes (KEGG) pathway and gene network analysis was performed. Functional protein association networks (STRING) analysis were performed at the highest confidence score to know the function-related protein-protein interactions. Clustering analysis and topological properties were analysed using Network Analyzer plugin of Cytoscape (3.5.1) [5].

### The Receptors:

Gene network analysis identified the important target gene in the apoptotic pathway for development of phytotherapeutics. The three-dimensional structures of the receptors were downloaded from RCSB-Protein data bank [www.rcsb.org/pdb] in .pdb format. The structures of both the receptors were determined by X-Ray diffraction with resolution of 2.20 Å and 2.67 Å respectively. The stereochemical characterization of the receptors was performed by generating Ramachandran plot using PROCHECK module of the PDBSum server [www.ebi.ac.uk/].

### The Ligands:

4 natural compounds were selected for docking with the two-receptor proteins and these were obtained from the QTOF-LC-MS of Acetone extract of O. alismoides. The SMILE structures of the compounds were downloaded from PubChem compounds database and converted to desired structure format with Open Babel for docking. Drug likeness property of the compounds was predicted with the online server Molsoft L.L.C. (www.molsoft.com).

### Molecular Docking:

Using FlexX of Biosolveit LeadIT software package, molecular docking was undertaken between the receptors and the ligands. The proteins were loaded into FlexX and receptor preparation was done for docking. The receptor preparation includes selection of protein chain, identification of pocket in the chain, water molecule removal and docking range selection in terms of radius involving amino acid residues. After preceptor preparation was confirmed, ligands structures were loaded, and the final docking was processed.

## Results and Discussion:

### Gene network analysis:

82 genes involved in the apoptotic pathway were retrieved from the KEGG pathway and gene network analysis was done. STRING analysis revealed 284 functional partners at the highest confidence score >0.9. Clustering analysis using MCODE algorithm of these functional partners showed 4 highly interconnecting clusters with 30 nodes (genes) (Figure 4). The first cluster consisting of 11 nodes was considered for further analysis. Topological properties, e.g., Average Shortest Path Length and Betweenness Centrality were analyzed using Network Analyzer Plugin of Cytoscape (3.5.1) to identify two genes, i.e., CFLAR and TRAF2 as the most influential genes in the apoptotic pathway.

### stereochemical quality assessment of the receptors:

Ramachandran plot were generated using PROCHECK module of the PDBSum server for stereochemical characterization of the receptors, which showed that 91.4% and 95% of the residues (169 and 342 residues) fall in the most favoured regions of the plot in CFLAR and TRAF2 respectively and it was also found that in both the receptors the percent of residue fall in disallowed region are 0%. Further the residues in additional allowed regions in the receptors are 8.6% and 5% (16 and 18 residues) respectively. The G-factors overall average score of the receptors are 0.29 and 0.11 respectively. This indicates the better stereochemical quality of the receptors for receptor-ligand interaction.

### Ligand-receptors interaction:

All the selected ligands were docked with both the receptors and it was found that all the ligands interacted with the CFLAR but a few of the ligand remains away from the interaction with the TRAF2. CFLAR have single peptide chain where as TRAF2 formed of 6 peptide chains and among them chain A has no active pocket for interaction. CFLAR contains 5 pockets for interaction with the ligand and among them pocket 1 contains 13 amino acid residues, pocket 2 and 4 contains 9 residue each, pocket 3 and 5 contains 8 and 7 residues respectively for hydrogen bond formation. Chain B of TRAF2 contains 1 pocket of 8 residues; chain C contains 3 pockets of 4 residue each. Chain D contains 5 pockets, chain E contains 5 pockets and chain F contains 4 pockets. The pockets were determined as active site of interaction with the ligands and those pockets contains larger number of amino acid residues have chance of getting more interaction with the ligand active site. Potent interaction has been seen among a few of the ligand with the receptors. Ottelione, Ropivacaine, Flupenthixol and Desmethylastemizole have shown good docking score with both the receptors. Ottelione formed 2 strong hydrogen bonds with the CFLAR along with some weak interaction, ropivacaine shown 1 strong hydrogen bond with 2 weak bonds; flupenthixol shown 2 strong hydrogen bond and 2 weak bond and desmethylastemizole contains 2 strong hydrogen bond and 1 weak bond.

## Discussion:

In modern day drug discovery approach, plant-based compounds have been delivering a great starting material in development of lead structure [6]. Phytochemicals are known to possess several therapeutics activities. The anti-cancer activity of plant compounds has also been reported in several experiments. The current computational study was conducted to identify phytotherapeutic compound for inhibition of proliferation function in cancer cells through inhibiting the activity of mutated CFLAR and TRAF2 function. Through gene network analysis of the apoptotic pathway gene in non-small cell lung carcinoma, CFLAR and TRAF2 were identified as major target as this two are the regulator of apoptosis [7]. Gene network analysis and string analysis are the two important tools in identifying the target receptor in insilico drug development process [5]. Molecular docking plays an important part in the drug discovery process as mechanism of action of the drug molecule can be identified based on inter molecular interaction among the receptor and the ligand [8]. The ligands molecule under study was taken from the LC-MS-QTOF of acetone extract of O. alismoides. All the ligands under this study possesses drug like characters predicted in Molsoft online server with drug-likeness model score of -0.01 to 1.77 (Table 1 - see PDF). Drug likeness of a compound is a primary criterion for the compound to be a drug [8]. The compounds ottelione, ropivacaine, flupenthixol and desmethylastemizole have shown good interaction with the receptors (Table 1-6 - see PDF). Desmethylastemizole showed better interaction with all the chain of TRAF2 than other compounds and in case of CFLAR, flupenthixol showed better interaction than others. The hydrogen bond among the flupenthixol and CFLAR formed of strong bond energy in compared with the other compounds and the receptor. The bonded atom of the ligand and the receptor are hydrogen and oxygen, which formed stronger affinity between the two bonding molecules.

**Figure 3:**
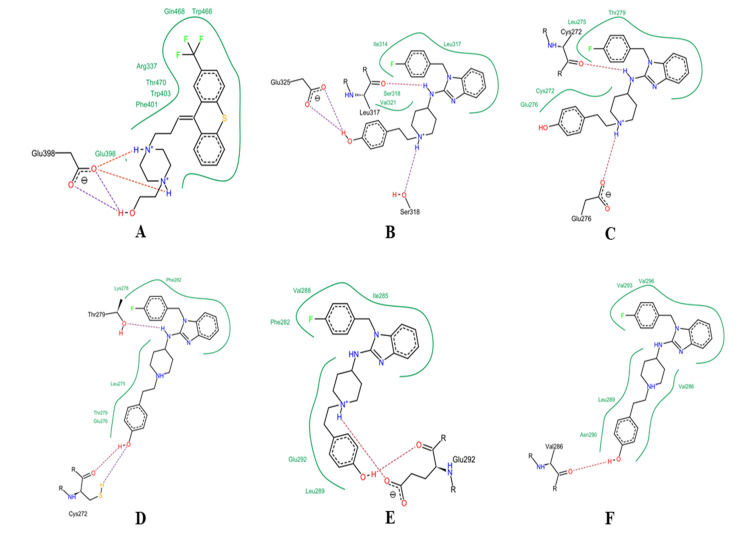
Bonding pattern among ligand and the receptor. A: Flupenthixol – CFLAR; B: Desmethylastemizole- TRAF2-B; C: Desmethylastemizole- TRAF2-C; D: Desmethylastemizole- TRAF2-D; E: Desmethylastemizole- TRAF2-E; F: Desmethylastemizole- TRAF2-F

**Figure 4:**
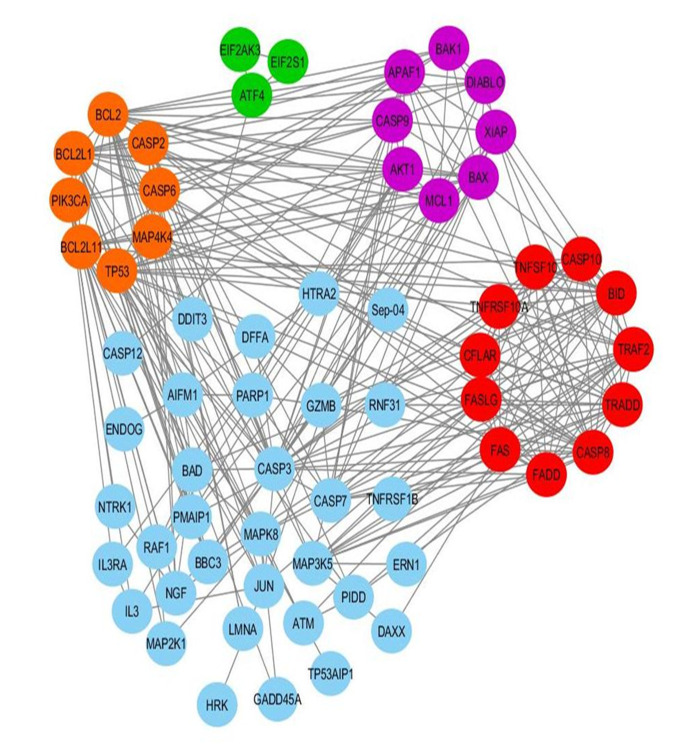
MCODE interconnected cluster generated from Cytoscape, showing two regulator genes TRAF2 and CFLAR in the network.

CFLAR and TRAF2 are two important regulator protein in the apoptotic pathway of a cell. Both the two proteins maintain the apoptotic pathway through proper signal transduction [9]. The mutation in CFLAR and TRAF2 destruct the apoptotic process and the cell follows the proliferation step and resulted to numerous numbers of cancer cells in the body. In the current study phytochemicals were identified to mitigate the ill effect of mutation in both the receptors so that the apoptotic pathway in the cell can be regenerated back to breakdown the process of cancer cell proliferation. CFLAR consist of only one polypeptide chain and 5 pockets, mutation in one of the pockets can alter its activity towards the proliferation process. In the molecular docking analysis, flupenthixol was found to interact with the active site of CFLAR more strongly among the ligands. And this inter-molecular interaction among the atom of ligand and amino acid residue of receptor in the active site inhibit the ill function of mutated CFLAR. The amino acid residue Glu398 of the receptor actively involved in the bonding with NH group of flupenthixol, in which 2 strong hydrogen bonds along with 2 weak interactions was formed. In the molecular interaction the docking score of -17.7859 was found with bond energy of -7.3 and -6.0. In TRAF2, out of six-polypeptide chain, chain A does not contain any active site of interaction with ligand, whereas chain B to chain F contains one or multiple number of pockets for interaction. In all the chain of TRAF2, desmethylastemizole showed more potent interaction with the active site of receptor, with docking score of -8.5489, -7.5664, -11.0232, -8.8325, -8.3772 in chain B to F respectively. Amino acid residue involved in the interactions is Glu325, Ser318, Glu276, Cys272, Thr279, Cys272, Glu292 and Val286 (Figure 3). The strong interactions portrayed by ligand on the mutated receptors inhibit proliferation signal transduction process in the cell. As a result of that inhibition the ill effect of mutation on the cell may be checked and the process of apoptosis may get its way back on the cell. Mutation plays a devastating part in the development of several cancer [7] and in the current study mutation in CFLAR and TRAF2, regulator of apoptotic pathway, disrupt the process of natural death in the cell. Finding of two natural compounds flupenthixol and desmethylastemizole showed potent inhibition activity through strong interaction to check the proliferation of cell.

## Conclusion:

We document the molecular docking analysis data of Flupenthixol and desmethylastemizole with the apoptotic regulator proteins CFLAR and TRAF2 linked to lung carcinoma for consideration.
